# A novel self-transmissible mega plasmid from extensively drug-resistant *Klebsiella oxytoca* carries multiple antimicrobial resistance genes and acts as a resistance reservoir

**DOI:** 10.1016/j.crmicr.2026.100587

**Published:** 2026-03-24

**Authors:** Yu Wang, Sylvia A. Sapula, Bradley J. Hart, Henrietta Venter

**Affiliations:** School of Pharmacy and Biomedical Science, College of Health, Adelaide University, Adelaide, Australia

**Keywords:** Multi-drug resistance, Hospital wastewater, Conjugative plasmid, Antimicrobial resistance, Resistance reservoir

## Abstract

*Klebsiella oxytoca* is an opportunistic pathogen linked to hospital-acquired infections, with extensively drug-resistant (XDR) strains posing serious treatment challenges. Here, an XDR *K. oxytoca* 611 isolated from hospital wastewater was found to carry 59 antimicrobial resistance genes (ARGs) compared to the average of six ARGs for *K. oxytoca*. Twenty-eight of these ARGs were on a mega hybrid plasmid (pKO611.1) carrying C, HI2 and HI2A replicons. Among the resistance genes carried on pKO611.1 was a new tetracycline efflux pump *tet*(E) variant and an uncharacterised AmpC β-lactamase gene. Cloning and characterising of these genes in *Escherichia coli* confirmed their ability to confer resistance to tetracyclines and β-lactams, respectively. Conjugation between *K. oxytoca* and *E. coli* transferred pKO611.1 with high efficiency. Propagation of plasmid pKO611.1 conferred multi-drug resistance to *E. coli*. Attempts to cure pKO611.1 from *K. oxytoca* produced a shortened version of the plasmid where a 63,815 bp segment, containing several resistance genes, was excised. Accordingly, *K. oxytoca* harbouring the shortened version of the plasmid, did not display resistance to the associated antibiotics. The ease of inter-genera transmission of pKO611.1 and its ability to recombine indicates its potential role as a reservoir for dissemination of multiple ARGs.

## Highlights

• XDR *Klebsiella oxytoca* found in hospital wastewater with 59 resistance genes.
• Mega plasmid pKO611.1 carries 28 resistance genes, enabling multidrug resistance.
• Novel *tet*(E) variant and AmpC β-lactamase confer tetracycline & β-lactam resistance.
• Plasmid pKO611.1 transfers efficiently to *Escherichia coli*, spreading resistance.
• Plasmid recombination shows potential as resistance gene reservoir for dissemination.

## Graphical abstract

**Figure:**
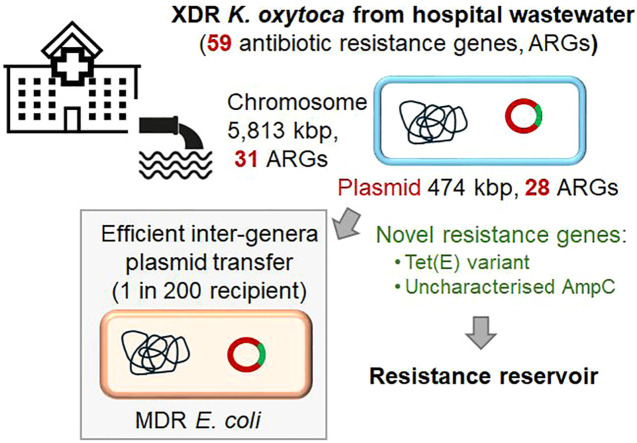

## 1 Introduction

*Klebsiella oxytoca* is an emerging pathogen known for causing both hospital and community-acquired infections. Although it is an environmental bacterium originating from water and soil, it is also a human commensal that can be found in the gut, oropharynx, and on the skin (Yang et al., 2022). When spread to other parts of the body, it can cause infections including urinary tract infections, antibiotic-associated haemorrhagic colitis, and bacteraemia, amongst others (Neog et al., 2021). Outbreaks of *K. oxytoca* have been reported worldwide, especially in intensive care units where patients are neonatal or critically ill (Yang et al., 2022; Liébana-Rodríguez et al., 2024).

The *K. oxytoca* complex comprises 9 closely related species, of which *K. michiganensis* and *K. oxytoca* carry more virulence genes and are the major species causing human infections (Li et al., 2024; Long et al., 2022). Among all *Klebsiella* spp., *K. oxytoca* is the second most clinically prevalent species after *K. pneumoniae* (Broberg et al., 2014). *K. oxytoca* isolates are frequently resistant to multiple commonly used antibiotics including penicillins, older generation cephalosporins, tetracyclines and quinolones (Moradigaravand et al., 2017; Ikhimiukor et al., 2023). Increasing, nonsusceptibility to last-resort antibiotics such as carbapenems, colistin, and tigecycline have also been observed, which is very concerning as it further limits treatment options for *K. oxytoca* infections (Yang et al., 2022).

Horizontal gene transfer (HGT) of antimicrobial resistance genes (ARGs) carried by mobile genetic elements is a growing issue which exacerbates the disease burden of *K. oxytoca* (Ikhimiukor et al., 2023; Stewart et al., 2022). Despite the increasing prevalence of extensively drug-resistant (XDR; defined as non-susceptibility to at least one agent in all but two or fewer antimicrobial categories (Magiorakos et al., 2012)) *K. oxytoca* associated nosocomial outbreaks (Larsen et al., 2025), surveillance data are lacking for this bacterial species. Moreover, *K. oxytoca* is ubiquitous in the environment and may potentially act as a vessel for ARG dissemination.

Healthcare environments, including hospital toilets and drainage systems, have been identified as reservoirs of multidrug resistant (MDR; resistance to one or more antibiotics in 3 different classes) bacteria (Larsen et al., 2025; Ludden et al., 2017; Sapula et al., 2024; Blaikie et al., 2024). These environments provide the opportunity for the exchange of genomic material by horizontal transfer. Hospital wastewater in particular is known as a hot spot for ARG dissemination, due to the presence of high concentrations of antibiotics and MDR bacteria in comparison to surface water (Ngigi et al., 2019). Among various HGT mechanisms, plasmid conjugation is the major driving force in bacterial evolution and an important contributor to the dissemination of ARGs across both animal-associated and environmental bacteria, for example, plasmid-encoded carbapenemases (NDM, IMP, KPC and OXA-48) have now been reported worldwide (Wan et al., 2024; Chen et al., 2022; Bosch et al., 2017; Partridge, 2018; Boyd et al., 2022).

Mobile genetic elements (MGEs) such as integrative and conjugative elements and bacteriophages (Tokuda and Shintani, 2024) also facilitate the horizontal gene transfer of ARGs, while transposons and insertion sequences facilitate the transmission of ARGs within bacterial genomes, thereby further accelerating the propagation of antibiotic resistance in clinical settings (Wang et al., 2024a). ARGs in plasmids can cross clinical and environmental boundaries, with wastewater serving as the ideal environment where antimicrobial resistance (AMR) emerges (Wang et al., 2024a; Berglund et al., 2023).

In this study, we report on the isolation of an XDR *K. oxytoca* from hospital wastewater in Adelaide, Australia. A combination of long- and short-read sequencing was used for the comprehensive analysis of its complete genome to investigate ARGs contributing to the XDR phenotype. We identified a novel hybrid plasmid, pKO611.1.1, which was further characterised for ARGs and its ability to transfer these via conjugation. This work highlights the role of polluted environments such as hospital wastewater in the origin and transmission of AMR and pinpoint pKO611.1 as a reservoir for resistance that can be transferred between different bacterial genera. Surveillance studies often overlook *K. oxytoca,* leading to the underestimation of its role as an important reservoir and disseminator of ARGs carried by MGEs such as pKO611.1.

## 2 Methods

### 2.1 Bacterial strains and reagents

*K. oxytoca* 611 was isolated from wastewater sampled from a hospital in Adelaide (Australia) as part of a project on the dissemination of AMR in 2018 (BioProject: PRJNA1018274). *E. coli* XL10-Gold (Agilent) was used for the creation of constructs and plasmid propagation. *E. coli* BL21(DE3) was used for phenotypic assays. Unless otherwise stated, all strains were grown aerobically at 37 °C in Difco™ Lysogeny Broth (LB) Miller (BD) supplemented with kanamycin 25 mg/L when necessary for maintaining pET41a(+) constructs. Antibiotics were purchased from Sigma-Aldrich and Glentham Life Sciences. Chemical reagents and oligonucleotides were purchased from Sigma-Aldrich. Enzymes used in cloning were purchased from New England Biolabs.

### 2.2 Cloning and construction of plasmids

The plasmid pET-41a(+) (Novagen) was used for the creation of constructs. Primers Tet(E)_*Nde*I_F 5′-GTTGTTCATATGAACCGCACTGTGATGATGGC-3′ and Tet(E)_*Xho*I_R 5′-GTTGTTCTCGAGAAGAAATGGCTGTGAATTGC-3′, AKO-1_*Nde*I_F 5′-GTCGTCCATATGATACCCAAGACGCTTTTC-3′ and AKO-1_*Bam*HI_His_R 5′-GTCGTCGGATCCTTAATGATGATGATGATGATGCTGTAAGGCGTTAAGAATGCG-3′ were used to amplify Hexa-His (6xHis) tagged *tet*(E) and *bla*_AKO-1_ from *K. oxytoca* 611. PCR products were digested with *Nde*I and *Xho*I or *Bam*HI (New England Biolabs) and ligated into pET-41a(+) to generate full-length constructs which were subsequently transformed into CaCl_2_ treated *E. coli* BL21(DE3) cells for phenotypic assays. Both constructs were confirmed by Sanger Sequencing (Australian Genome Research Facility).

### 2.3 Antimicrobial susceptibility testing

The *in vitro* minimum inhibitory concentrations (MICs) of a full panel of antibiotics and biocides were determined using broth microdilution, as previously described (Wang et al., 2024b) and according to the International Standard ISO 20,776–1 as recommended by EUCAST (the European Committee on Antimicrobial Susceptibility Testing). The results were interpreted according to available EUCAST breakpoints (EUCAST 2024). *K. oxytoca* with MICs above the breakpoints was defined as resistant to the respective antibiotic and as sensitive for those equal or below breakpoints (EUCAST 2024; Morrissey et al., 2014). *E. coli* ATCC 25922 was included as a quality control strain.

*E. coli* BL21(DE3) cells carrying constructs were grown to an OD_600_ around 0.6 and induced using 0.1 µM Isopropyl-β-d-thiogalactopyranoside (IPTG). Cells were induced for 2 h before being added to 96-well plates containing antibiotics supplemented with 0.1 µM IPTG. OD_600_ readings were taken after overnight culture at 37 °C with shaking.

### 2.4 DNA extraction and whole genome sequencing

Genomic DNA was extracted from an overnight culture of *K. oxytoca* 611 using an MN NucleoSpin® Microbial DNA extraction kit (Machery-Nagel, Australia) following manufacturer’s instructions. Whole-genome sequencing (WGS) was carried out at SA Pathology (Adelaide, Australia) using the Illumina NextSeq 550 platform with the NextSq 500/550 Mid-Output kit v2.5 (300 cycles) (Illumina Inc, USA). Sequencing reads were assembled and annotated using SPAdes and Prokka as part of the TORMES pipeline v.1.3.1 (Quijada et al., 2019). Long-read WGS was performed using the Nanopore MinION Mk1B (Oxford, United Kingdom). MinION libraries were prepared using the rapid barcoding kit 24 V14 and then added to a MinION flow cell (R10.4.1) for sequencing. lllumina short reads and Nanopore long reads were used as input data for hybrid genome assembly using Hybracter (Bouras et al., 2024). Genome polishing was performed using Polypolish (Wick and Holt, 2022) and Polca (Zimin and Salzberg, 2020), and assessed using ALE (Clark et al., 2013). The TORMES pipeline was used again for the assessment of completeness and contiguity using Quast (Gurevich et al., 2013). Annotation was performed using Prokka v1.14.5 (Seemann, 2014) as part of the TORMES pipeline (Quijada et al., 2019).

### 2.5 Assessment of efflux pump activity using the checkerboard titration assay

The efflux of tetracyclines (tetracycline, doxycycline, minocycline, and tigecycline) was determined with checkerboard titration assays as described previously (Guo et al., 2023). Briefly, antibiotics were serially diluted vertically down a 96-well plate (Costar®) and the efflux pump inhibitors (EPIs), phenylalanine-arginine β-naphthylamide (PAβN) or Carbonyl Cyanide 3-Chlorophenylhydrazone (CCCP) were serially diluted horizontally across the same plate. A bacterial inoculum 5 × 10^5^ CFU/well was added into each well and incubated at 37 °C. OD_600_ readings were taken on a Cytation5® (Bio-Tek®) plate reader after 18±2 h. For compounds showing high absorbance at time zero, bacterial growth was assessed by adding 10 μL of 0.01 % resazurin, which was followed by incubation at 37 °C for 30 mins with shaking. The addition of resazurin results in metabolically active bacteria appearing pink while a blue colour indicates no bacterial growth.

### 2.6 Nitrocefin assay

A nitrocefin hydrolysis assay was performed on whole cells to confirm β-lactamase activity of the AKO-1 construct. Briefly, *E. coli* BL21(DE3) cells expressing the pET-41a(+)-*bla*_AKO-1_ were inoculated from an overnight culture in 50 mL of LB broth supplemented with 25 mg/L kanamycin. Inoculants were grown at 37 °C until OD_600_ reached 0.5. Cells were harvested at 5000 x *g* for 5 min at 4 °C. Cell pellets were washed three times and resuspended in KPi buffer (50 mM potassium phosphate buffer, pH 7.0). The desired concentrations of nitrocefin (dissolved in DMSO initially and diluted using KPi buffer) were placed in a 96-well plate (Costar®). Cells were added into wells to start the reaction. *E. coli* BL21(DE3) carrying the empty pET-41a(+) was used as control. Hydrolysis of nitrocefin was monitored by measurement of absorbance at 486 nm using a Cytation5® (Bio-Tek®) plate reader. Assays were performed independently on three different days.

### 2.7 Conjugation assay

*E. coli* BW25113 and *K. oxytoca* 611 cells were used as recipient and donor, respectively. Bacterial cells were inoculated from an overnight culture in LB broth and grown at 37 °C until OD_600_ 0.6–0.8. Cells were collected by centrifugation at 4000 x *g* for 5 min (1 mL of recipient and donor cells separately). Cells were washed twice in phosphate buffered saline (PBS) and reconstituted in 1 mL LB broth. Recipient and donor cultures (30 µL of each; ratio 1:1) were mixed and added to a circular sterile 0.2 µM cellulose nitrate filter (Satorius, Germany) and incubated on LB agar overnight at 37 °C. The filter was placed in 5 mL of PBS in a 50 mL tube using aseptic techniques. Bacteria on the filter paper were collected by gentle vortexing. Cells were centrifuged at 4000 x g for 5 min and washed twice in PBS solution. Serial dilutions of cells were then spread plated on Brilliance^TM^ *E. coli*/coliform selective agar (Oxoid, United Kingdom) with or without tetracycline (16 mg/L) before being incubated overnight at 37 °C. On this agar *Klebsiella* are a pink colour and *E. coli* appear as purple colonies, which were picked. *E. coli* transconjugants were confirmed by colony PCR using *Taq* polymerase (New England Biolabs, USA) and primers for *tet*(E) and *bla*_AKO-1_ as stated above. Matrix-assisted laser desorption ionization-time of flight (MALDI-TOF) mass spectrometry (Bruker Daltonik GmbH, Germany) was used to further confirm the identification of *E. coli* transconjugants. Conjugation frequency was calculated as the number of transconjugants growing on the tetracycline selection plate divided by the number of total recipient *E. coli* growing on the non-selective plate, based on conjugation assays performed on three different days.

### 2.8 Plasmid curing assay

Plasmid curing was performed using the intercalating dye acridine orange, following a previously described method (Trevors, 1986). Briefly, a 96-well plate was set up by serially diluting acridine orange from 200 to 0.5 µg/mL in LB broth. An overnight culture of *K. oxytoca* 611 was diluted and added into the plate at 5 × 10^5^ CFU/well followed by overnight incubation at 37 °C with shaking. Wells displaying bacterial growth at the highest concentration of acridine orange (200 µg/mL) were used, and samples were diluted and plated on LB agar. Fifty colonies were patched on LB agar supplemented with 32 µg/mL tetracycline. As all colonies were able to grow on an tetracycline agar plate, further assessment of the plasmid-cured isolates was performed using colony PCR. The isolate lacking the amplicons of two target genes (*tet*(E) and *bla*_AKO-1_) was analysed further using an antimicrobial susceptibility assay.

### 2.9 Bioinformatic analysis

Multiple protein sequence alignments were performed using Clustal Omega (Madeira et al., 2022) and visualized by the ESPript 3.0 server (Robert and Gouet, 2014). Plasmid analysis was performed and visualized using Proksee (Grant et al., 2023), where resistance genes (Alcock et al., 2023) were identified by the Comprehensive Antibiotic Resistance Database (CARD) and prophage regions were identified using Phigaro 2.3.0 (Starikova et al., 2020). Virulence factors were identified using a blast search against the VFDB core dataset (genes associated with experimentally verified virulence factors (http://www.mgc.ac.cn/VFs/) (Altschul et al., 1997). Incompatibility group and relaxase MOB family of plasmids was determined using PlasmidFinder 2.1 (Carattoli et al., 2014) and MOBscan (Garcillan-Barcia et al., 2020), respectively. To predict whether pKO611.1 is conjugative, CONJScan (Cury et al., 2020) was used to identify the conjugation system. HOTSPOT was used to predict the plasmid host (Ji et al., 2023). Furthermore, to identify insertion sequences, IS finder (Siguier et al., 2006) was used. Basic local alignment search tool (BLAST) analysis (Altschul et al., 1990) were performed to search for similar nucleotide or protein sequences. The signal peptide cleavage site of AmpC was predicted using SignalP 6.0 (Teufel et al., 2022). Sequence comparisons were performed using the β‑Lactamase DataBase BLAST tool, and the 50 annotated AmpC β‑lactamases yielding the most significant alignments were subsequently retrieved (Naas et al., 2017). A phylogenetic tree was generated using the maximum likelihood method and tested with the bootstrap method (1000 replicates) in MEGA12 (Molecular evolutionary genetic analysis) (Kumar et al., 2024). TMHMM 2.0 was used to predict transmembrane helices in Tet(E) (Krogh et al., 2001).

## 3 Results

### 3.1 *Klebsiella oxytoca* 611 is an extensively drug-resistant (XDR) bacterium conferring high levels of resistance against tetracycline and β-lactams

*K. oxytoca* 611 was isolated in 2018 from hospital wastewater in Adelaide (South Australia). Antimicrobial susceptibility testing revealed that this isolate was resistant to multiple classes of antibiotics including β-lactams (penicillins, cephalosporins, and monobactam), fluoroquinolones, aminoglycosides, chloramphenicol and tetracyclines including tigecycline, the last-resort antibiotic for the treatment of XDR Enterobacterales but remained susceptible to colistin and carbapenems (Table 1) thus was characterised as an XDR bacterium.

**Table 1:** Minimum inhibitory concentrations (MIC) of a panel of antimicrobials.

Green: Sensitive, susceptible, increased exposure or Wild-type; Beige: Resistant or Non-wild-type.

*EUCAST clinical breakpoint Version 15.0 2025 for Enterabacterales, if the MIC is more than this value, the strain is resistant to the antibiotic. Trimethoprim: sulfamethoxazole in the 1:19 ratio. Breakpoint and MICs are expressed as the trimethoprim concentration. There are some exceptions: Cefepime: Resistant (>4), Sensitive (≤1) Susceptible with increased exposure (>1 and ≤4).

**EUCAST does not provide a breakpoint for some tetracyclines and biocides for Enterobacterales, thus, ECOFFs (Epidemiological cut-off values) of tetracyclines are reported here to determine whether the isolate is wild type (WT) or non-wild type (NWT); ECOFFs of biocides for *Klebsiella* were obtained from Morrissey *et al*., 2014.

AMP, ampicillin; AZT, aztreonam; BLK, benzalkonium; CETR, cetrimide; CLE, cefalexin; CEP, cefepime; CHL, chloramphenicol; CHX, chlorohexidine; CTA, cefotaxime; CXI, cefoxitin; CTZ, ceftazidime; CIP, ciprofloxacin; COL, colistin; GEN, gentamicin; IMI, imipenem; LEV, levofloxacin; MER, meropenem; NA, Not Available; OXA, oxacillin; PIP, piperacillin; RIF, rifampicin; TCS, triclosan; TRS, trimethoprim-sulfamethoxazole; TIG, tigecycline; TOB, tobramycin.

### 3.2 Whole genome sequencing of *K. oxytoca* 611 revealed a novel hybrid plasmid carrying numerous ARG and multiple prophage regions

**Fig. 1:**
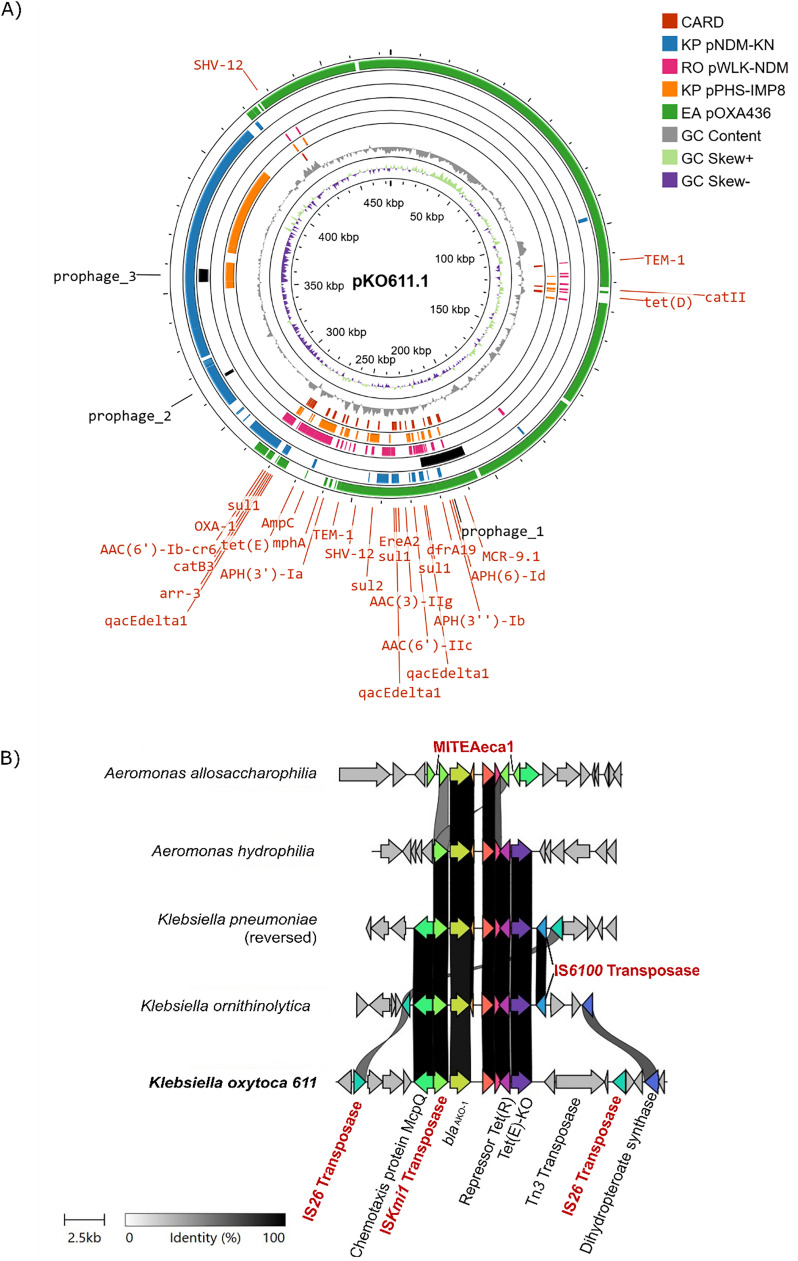
Schematic map of A) pKO611.1 (473,763 bp), B) transfer of resistance regions among bacterial species. **A)** From the outermost to the innermost, the rings represent the sequences that are shared between *Enterobacter asburiae* pOXA436 (GenBank KY863418.1) (green), *Klebsiella pneumoniae* pNDM-KN (GenBank JN157804.1) (blue), *R. ornithinolytica* pWLK-NDM (GenBank CP038280.1) (magenta), *Klebsiella pneumoniae* pPHS-IMP8 (GenBank OM975890.1) (orange). Also indicated are prophage regions (black), AMR genes (red), GC content (gray), and GC skew (+: Light green, -: Purple). **B)** Schematic presentation of the gene features of the resistance region in pKO611.1, in comparison with resistance regions in related plasmids carried by *Aeromonas allosaccharophilia* (GenBank CP118989.1), *Aeromonas hydrophilia* (GenBank CP028567.1), *K. pneumoniae* (GenBank OM975890.1), and *R. ornithinolytica* (GenBank CP038280.1). Areas shades in black indicate identity percentage. Direction of arrows shows the translation direction of ORFs.

Both short-read sequencing (Illumina) and long-read sequencing (Nanopore) were utilised to generate a complete sequence of *K. oxytoca* 611. *K. oxytoca* 611 belongs to sequence type (ST) 58 and contains a circular 5813,412 bp chromosome (GC content: 55.24 %) and two large plasmids with lengths of 473,763 bp (pKO611.1) and 208,440 bp (pKO611.2) respectively. HOTSPOT predicted the host of the larger plasmid being *Enterobacter hormaechei*, and the other being *K. pneumoniae*. Resistance gene analysis revealed a total of 59 resistance genes of which 31 were carried on chromosome, and 28 by pKO611.1 **(Table S1 and S2**) and none by pKO611.2. Virulence gene analysis revealed multiple virulence proteins chromosomally encoded (**Table S3**) and one encoded on pKO611.1 (**Table S4**). Comparative genomic analyses indicated that pKO611.1 shares no significant sequence, structural, or organisational similarity with any previously characterised plasmid (Fig. 1A). pKO611.1 carries C, HI2 and HI2A replicons and a MOB_H_-type relaxase, indicating its potential for conjugative transfer. It also revealed that pKO611.1 carried genetic material from plasmids belonging to both *Enterobacter asburiae* pOXA436 and *Klebsiella pneumoniae* pNDM-KN (Fig. 1A). However, a region of around 30 kbp, which carries multiple ARGs, does not appear in either the *E. asburiae* or *K. pneumoniae* plasmids. Parts of this region are carried by plasmids that originate from other bacteria including *K. pneumoniae* pPHS-IMP8 and *Raoultella ornithinolytica* pWLK-NDM. These two plasmids are much smaller in size, but both have a high average nucleotide identity (ANI) (84,438 bp and 97.7 % for pPHS-IMP8, 75,415 bp and 93.31 % for pWLK-NDM) when compared to pKO611.1. Interestingly, the *K. pneumoniae* plasmid, pPHS-IMP8, may potentially have emerged from the deletion and rearrangement of pKO611.1 as almost the full plasmid sequence can be mapped to pKO611.1 with high similarities.

In addition to ARGs, three prophage regions were identified on pKO611.1, with the largest (Prophage_1 in Fig. 1A) carrying ARGs including *dfrA19* and *mcr-9.1* amongst others **(Figure S1)**. A sequence similarity search using BLAST revealed that the same prophage region was also identified in two plasmids. One plasmid was detected in an *E. asburiae* which was isolated from a urine sample collected in 2013 from a Danish hospital (Samuelsen et al., 2018). Another plasmid was detected in an *Enterobacter hormaechei* isolated from a cervical swab of an expectant mother in Germany (Yao et al., 2022). Furthermore, this prophage region was also detected in a new species of *Enterobacter, Enterobacter adelaidei* with a 90 % coverage and 100 % identity. This strain was also isolated from hospital wastewater in Adelaide, in 2020 (Siderius et al., 2024).

Finally, pKO611.1 harbours many insertion sequences (IS), with ISfinder identifying over 30 types of IS and transposons showing greater than 95 % identity to known sequences. Notably, IS*26* was one of the most abundant elements identified on pKO611.1 **(Figure S2A)**. IS*26* belongs to the IS*6* family, is usually found on AMR plasmids (Snaith et al., 2023) and plays a critical role in ARG dissemination (Harmer and Hall, 2019).

### 3.3 Acridine orange treatment shortened pKO611.1

To investigate the role of pKO611.1 in resistance of *K. oxytoca* 611, acridine orange (AO) was used to cure plasmids from this isolate. Cells which had lost the plasmid were screened for by negative selection-replica plating on LB agar supplemented with tetracycline (32 μg/ml) and PCR amplification of *tet*(E) and *bla*_AmpC_ carried on pKO611.1, to screen for the loss of these genes. One of the isolates selected for AO-treatment and antibiotic susceptibility testing revealed a loss of resistance to aminoglycosides, rifamycin, and trimethoprim-sulfamethoxazole, but retained resistance to other antibiotic classes including β-lactams, tetracyclines, and chloramphenicol (Table 1). To further investigate whether AO-treatment successfully cured the plasmid, Nanopore sequencing was performed on the cured isolate, and a plasmid smaller than pKO611.1 (409,925 bp instead of the original 473,763 bp) was obtained, however, it was found to be missing most of the ARGs (**Figure S2A**). The remaining ARGs including those encoding β-lactamases (*bla*_SHV-12_, *bla*_TEM-1_), chloramphenicol resistance gene (*catII*), tetracycline efflux pump (*tet*(D)), and aminoglycoside phosphotransferase (*strB* and *strA*) correlated with the resistance observed from antibiotic susceptibility results. Further investigation revealed two identical Tn*21*_partial transposons on the same DNA strand flanking the missing region and a copy of a partial Tn*21* in the missing region (**Figure S2B**). As both partial Tn*21* still remained in the truncated plasmid and a third copy was present in the missing region, this deletion may potentially be due to homologous recombination which allowed for the deletion of a large region.

### 3.4 pKO611.1 confers high level resistance to *E. coli*

pKO611.1 appeared to carry a functional conjugative system, a F-Type IV secretion system (T4SS) with all three mandatory genes (T4SS_virb4, T4SS_t4cp, and MOB_H_) present. A conjugation assay was performed to examine whether pKO611.1 is truly conjugative. Following conjugation, PCR confirmed successful and highly efficient conjugation to *E. coli* BW25113 with a frequency of conjugation of 0.5 × 10^2^ per recipient cell. Five colonies were picked from the plate and tested for MICs. Results revealed that all these isolates were resistant to all β-lactams (4- to 1024-fold increase in MICs) except for carbapenems, tetracyclines (4- to 128-fold increase), aminoglycosides (16- to 64-fold increase) and chloramphenicol (>8-fold increase in MICs) indicating that pKO611.1 and the AMR genes it carries were able to confer resistance to this *E. coli* through conjugation (Table 1). One of the transconjugants was further confirmed by Oxford Nanopore sequencing.

Finally, growth of *K. oxytoca* 611 and *K. oxytoca* (AO-treated) was assessed, and no fitness changes were observed between these two strains. There was also no fitness cost observed for the *E. coli* transconjugant BW25113+pKO611.1 when compared with the original *E. coli* BW25113 (**Figure S3**).

### 3.5 The pKO611.1 resistance region contains an uncharacterised AmpC, a new variant of the Tet(E) efflux pump, and a virulence factor

pKO611.1 carries a 7946 bp resistance region harbouring *tet*(E) and *bla*_AmpC_ which encode a Tet(E) efflux pump and an AmpC β-lactamase. Homologues of both genes are present in the chromosome of *Aeromonas veronii* (CP087266), where *bla*_AmpC_ is disrupted by an IS*As22* transposon insertion. Regions with similarity to the pKO611.1 resistance region were also identified in plasmids carried by *R. ornithinolytica, K. pneumoniae*, and *Aeromonas hydrophila* (Fig. 1B). In a plasmid from *A. allosaccharophilia, bla*_AmpC_ was flanked by two copies of IS*Kmi1* (IS*30* family). However, the IS*Kmi1* elements were disrupted by the insertion of MITEAeca1. A blast search of the IS*Kmi1*-flanked *bla*_AmpC_ region revealed that this region was only found in *A. allosaccharophilia*. For *tet*(E), the right inverted repeat (IRR) of Tn6433 (5′-GGGGGAACCGCAGAATTCGGAAAAAATCGTACGCTAAG-3′), a recently described transposon involved in HGT in *Aeromonas* spp. was identified whereas the left inverted repeat (IRL) was not detected (Shi et al., 2020). Collectively, these findings show that this resistance gene cluster occurs across multiple plasmids associated with Gammaproteobacteria. Additionally, this region also harbours a virulence factor gene, *mcpQ*, encoding a methyl-accepting chemotaxis protein (MCP).

### 3.6 The uncharacterised AmpC conferred β-lactam resistance to *E. coli*

**Fig. 2:**
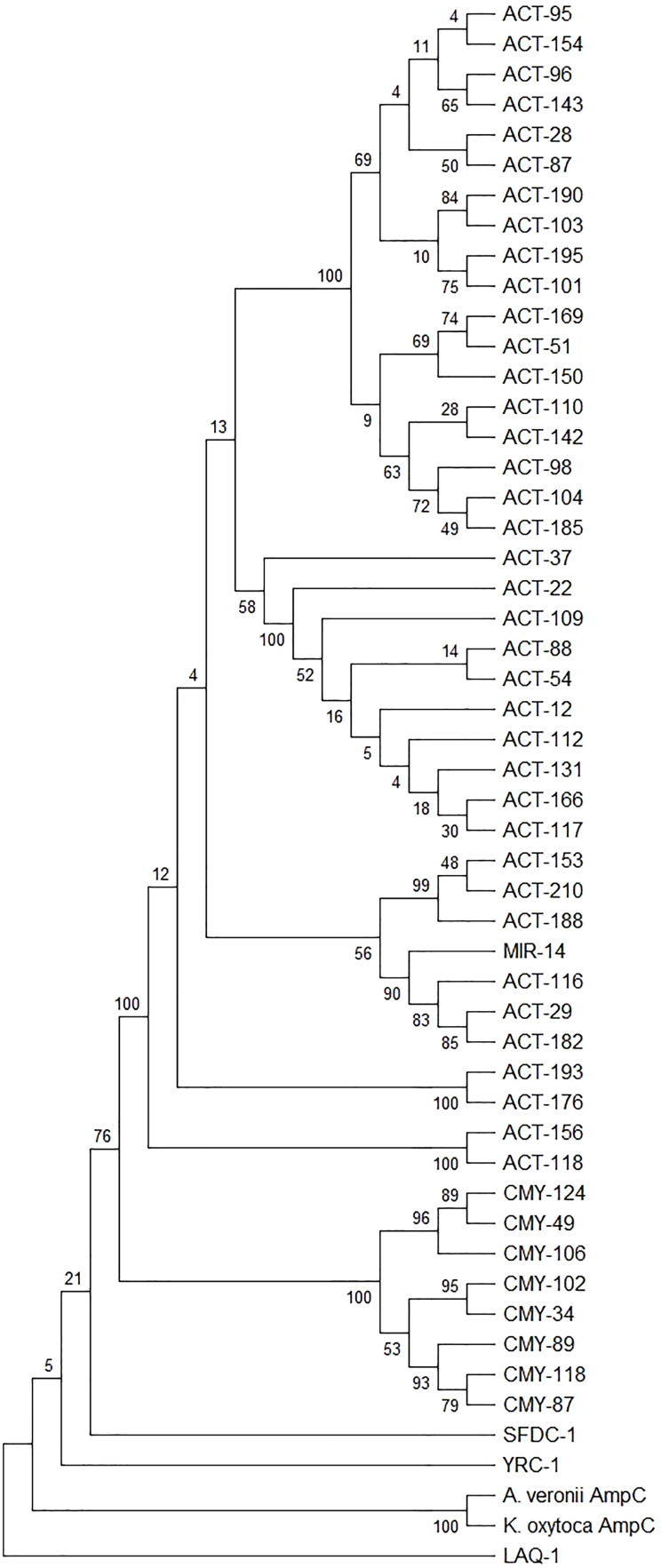
Phylogenetic tree of the Blast search result of the 50 sequences of highest homology using the BLDB (β-lactamase database). The maximum likelihood phylogenetic tree was constructed using MEGA12. Numbers shown are bootstrap frequency (1000 bootstrap).

The exact *bla*_AmpC_ gene found on pKO611.1 is 99.83 % identical to a *bla*_A__mpC_ gene from *A. veronii, A. allosaccharophilia, A. hydrophilia, K. pneumoniae*, and *R. ornithinolytica*. In total, five protein variants encoded by different alleles were identified (**Figure S4**). However, none of these closely related AmpC β-lactamases have been characterized before, thus the resulting AmpC β-lactamase was designated AKO-1 (for AmpC *K. oxytoca*). Phylogenetic analysis indicated that AKO-1 clustered with the chromosomal AmpC enzymes from *Aeromonas* spp. with strong bootstrap frequency (100) and forming a distinct lineage separate from previously described AmpC families including ACT, CMY, MIR, SFDC, and YRC (Fig. 2). Together with the high amino acid identify (99.83 %) to the *Aeromonas* spp. AmpC and the genetic mobilization analysis (Fig. 1**B**), these results suggest that the plasmid-encoded AmpC likely originated from an *Aeromonas* chromosome and may represent a novel mobilized AmpC family.

**Fig. 3:**
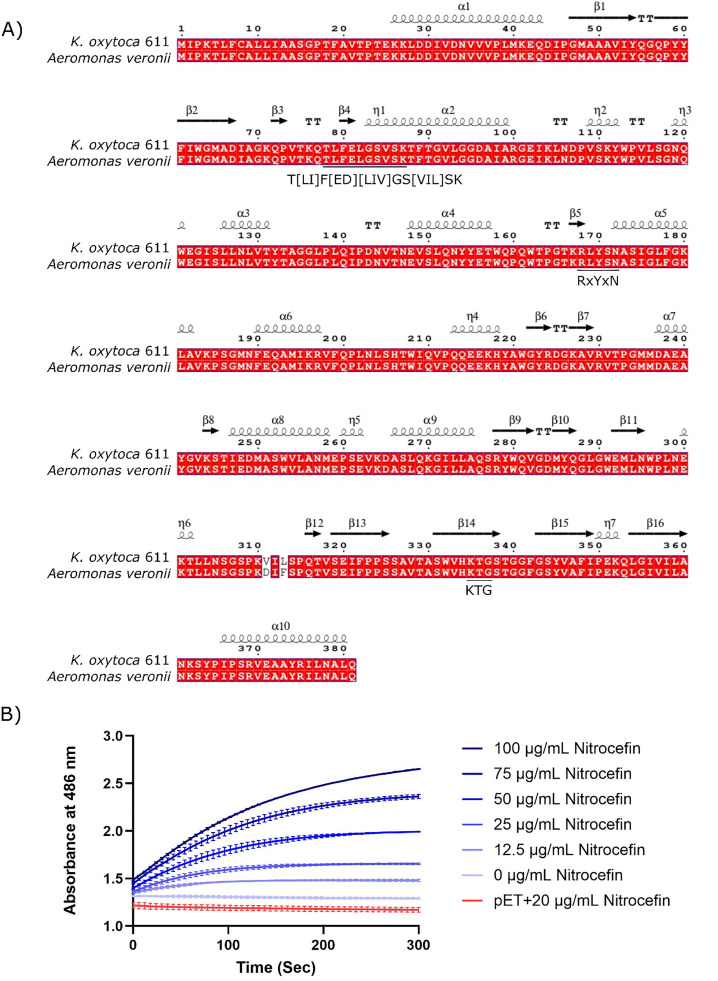
**A)** Amino acid alignment of AKO-1 and an *A. veronii* AmpC (Accession number: WP_101,618,176.1). Secondary structure was predicted based on the crystal structure of AmpC (PDB ID: 4KG2) from *E. coli*. The cleavage site of the signal peptide is denoted by a triangle. Conserved motifs are underlined and indicated. **B)** Hydrolytic activity for different concentrations of nitrocefin was detected at 486 nm. Cell only controls and vector controls were included. Points represent the mean ± standard error of the mean (SEM).

AKO-1 was further assessed by an amino acid sequence alignment with the closely related AmpC from *Aeromonas* spp. (Fig. 3**A**). Two amino acid differences, V311D and L313F, are present proximal to the C-terminal end. Assessment of known conserved regions identified in AmpC serine β-lactamases revealed that AKO-1 has all of the conserved motifs (T[LI]F[ED][LIV]GS[VIL]SK, RxYxN and KTG) responsible for catalytic activity or substrate binding (Brandt et al., 2017) (Fig. 3A). Further analysis also revealed that the signal peptide of the protein was predicted to be cleaved between alanine (A20) and valine (V21), by signal peptidase I, as β-lactamases are secreted into the periplasmic space where the β-lactams act.

*bla*_AKO-1_ was cloned and expressed in *E. coli* BL21(DE3) as a recombinant protein propagated by pET-41a(+). An antibiotic susceptibility assay of this construct revealed that it conferred resistance upon *E. coli* to penicillins, cephalosporins including the last generation cefepime and the monobactam, aztreonam (Table 2). Furthermore, β-lactamase inhibitors including clavulanic acid and tazobactam provided no inhibitory effects against AKO-1 which is commonly observed with AmpC β-lactamases (Table 2).

**Table 2:** Minimum inhibitory concentrations (MIC) against *E. coli* BL21(DE3) expressing AKO-1.

Antibiotic class	Antibiotic	*E. coli* BL21-pET-41a(+)	*E. coli* BL21(pET- *bla*_AKO-1_)	Fold-change of MIC
		MIC (μg/mL)		
**Penicillins**	AMP	1	8	8
	PIP	0.5	2	4
**Penicillin + *B*-lactamase inhibitor**	AMP+TAZ	1	8	8
	PIP+TAZ	0.5	2	4
**Cephalosporins**	CLE	4	128	32
	CXI	0.0625	2	32
	CTA	<0.016	1	>64
	CTZ	0.0625	2	32
	CEP	<0.016	0.03	>2
**Cephalosporin + Β-lactamase inhibitor**	CLE+CLV	4	128	32
	CLE+TAZ	4	64	16
**Carbapenems**	IMI	1	1	-
	MER	0.03125	0.03125	-
**Monobactam**	AZT	0.00781	0.0625	8

AMP, ampicillin; AZT, aztreonam; CLV, clavulanic acid; CLE, cefalexin; CEP, cefepime; CTA, cefotaxime; CXI, cefoxitin; CTZ, ceftazidime; IMI, imipenem; MER, meropenem; PIP, piperacillin; TAZ, tazobactam.

*CLV at 2 µg/mL, TAZ at 4 µg/mL.

AKO-1 β-lactam hydrolytic activity was also assessed using the chromogenic cephalosporin, nitrocefin in *E. coli* BL21(DE3) cells carrying this construct. β-lactamase activity is observed as an increase in absorbance at 486 nm. The rate of nitrocefin hydrolysis displayed a dose dependent increase (Fig. 3B).

### 3.7 The *tet*(E) variant originated from *Aeromonas* and confers tetracycline resistance to *E. coli*

Sequencing analysis of *K. oxytoca* 611 revealed multiple chromosomally encoded RND efflux pumps (**Table S2**) in addition to tetracycline efflux proteins which were encoded on pKO611.1 (**Table S1**). The presence of these pumps explains the high levels of tetracycline resistance observed for this isolate. To determine the contribution of these pumps to tetracycline resistance, an inhibition assay was carried out. Two different efflux pump inhibitors (EPIs) were used in this analysis, Phenylalanine-Arginine Β-Naphthylamide (PAβN), a known RND EPI (Venter et al., 2015) and carbonyl cyanide 3-chlorophenylhydrazone (CCCP) (Ardebili et al., 2014), a non-specific disruptor of efflux activity. Addition of PAβN resulted in a 64-fold reduction in minocycline MIC and a 2-fold reduction for other tetracyclines (Table 3). Addition of CCCP, reduced the MICs of tetracycline and doxycycline 16-fold, while 4- to 8-fold reductions were observed for minocycline and tigecycline. These results demonstrate that RND efflux pumps are potentially the main contributors of minocycline resistance in *K. oxytoca* 611, but other efflux pump types, such as the MFS efflux pump Tet(E) may play the main role in tetracycline and doxycycline resistance.

**Table 3:** Susceptibility to tetracycline antibiotics.

	Minimum Inhibitory Concentration (μg/mL) (fold-change relative to MIC of controls with no EPIs are indicated in brackets)				
Antibiotics	*K. oxytoca* 611			*E. coli* BL21 (pET-41a(+))	*E. coli* BL21(pET-Tet(E)-KO)
	With no EPIs	With 128 μg/mL of PAβN	With 40 μg/mL of CCCP		
TET	512	256 (2)	32 (16)	0.5	32 (64)
DOX	64	16 (4)	4 (16)	0.25	4 (16)
MIN	128	2 (64)	16 (8)	0.25	1 (4)
TIG	4	1 (4)	1(4)	0.25	1 (4)

DOX, Doxycycline; MIN, Minocycline; TET, Tetracycline; TIG, Tigecycline.

BLASTp results revealed that Tet(E) is widely distributed among gammaproteobacteria, including *Aeromonas* spp., *E. coli, Salmonella enterica,* and *Vibrio* spp. However, this variant has not been characterised and was designated Tet(E)-KO. Tet(E)-KO is predicted to contain 12 transmembrane helices (TMS), typical of other Tet(E) pumps. Sequence alignment revealed that Tet(E)-KO is closely related to the Tet(E) (WP_011899270.1) with 99.75 % similarity and 98.77 % identity. Tet(E)-KO contains all 12 MFS TMS motifs (**Figure S5**).

The ability of Tet(E)-KO to confer resistance against tetracyclines was assessed in *E. coli* BL21(DE3) cells. It was able to confer high levels of tetracycline resistance to *E. coli* (up to 64 x the MIC of the control strain harbouring empty pET-41a(+) (Table 3). It was also able to confer resistance against later generation tetracyclines, including minocycline, doxycycline and the last resort antibiotic, tigecycline.

## 4 Discussion

MDR *K. oxytoca* is frequently isolated from healthcare wastewaters, where it disseminates and acquires ARGs and actively contributes to the development of AMR (Larsen et al., 2025; Ikhimiukor et al., 2023). These features make *K. oxytoca* a significant threat to human health. In this study, we isolated XDR *K. oxytoca* KO611 from hospital wastewater. KO611 was resistant to all antibiotics tested except for carbapenems and colistin. Notably, KO611 was resistant to the fourth-generation cephalosporin cefepime, and tigecycline, a last-resort antibiotic used for the treatment of MDR Enterobacterales (Kelesidis et al., 2008).

Whole genome sequencing revealed that *K. oxytoca* 611 carried 59 ARGs, of which 28 are on a novel plasmid, designated pKO611.1. In comparison, an analysis of 324 genomes of clinical *K. oxytoca* isolates found these organisms carry an average of only six ARGs with 25 being the maximum number of AGRs observed (Ikhimiukor et al., 2023). In addition to its high ARG content, pKO611.1 is also very large (473,763 bp) and carries C, HI2 and HI2A replicons, suggesting that it is a hybrid. IncC plasmids were initially isolated from *Providencia stuartii, Pseudomonas aeruginosa*, and *Klebsiella pneumoniae* in the late 1960s and subsequently have been found in other Gram-negative bacteria including *S. enterica, E. coli* and *Vibrio cholera* (Ambrose et al., 2018). IncC plasmids are also commonly found in MDR Enterobacterales and have become one of the most widely reported plasmid types associated with AMR globally (Wang et al., 2015; Cao et al., 2015; Rozwandowicz et al., 2018). In addition, IncHI2 plasmids are also known to carry numerous ARGs, and are usually conjugative, especially among enteric bacteria (Algarni et al., 2024). Carrying C, HI2 and HI2A replicons means that pKO611.1 has a potentially broad host range. Moreover, larger plasmids generally carry more ARGs and are more likely to carry extended-spectrum β-lactamases and carbapenemases compared to smaller plasmids (Orlek et al., 2023). pKO611.1 also carries numerous IS elements belonging to the IS*6* family. The presence of these promotes the acquisition of ARGs as exemplified by the mobilisation of the resistance region analysed in this study.

The high levels of resistance observed for *E. coli* transconjugants against β-lactams, tetracyclines, aminoglycosides, chloramphenicol, rifampicin, and trimethoprim-sulfamethoxazole shows the capability of pKO611.1 to confer an XDR phenotype across species and genera. Although the *E. coli* transconjugant was not resistant to cefepime or tigecycline, the MIC values were very close to the clinical breakpoints. The plasmid could confer cefepime and tigecycline resistance to bacteria that already have low level of resistance due to other resistance factors such as porin mutations (Uehara et al., 2025).

Attempts to cure pKO611.1 from *K. oxytoca* 611 using high concentrations of AO resulted in a truncated version of pKO611.1. The isolate carrying the truncated pKO611.1 remained resistant to most of the antibiotics assessed with the exception of aminoglycosides, rifamycin, and trimethoprim-sulfamethoxazole. Although the addition of AO did not result in curing of pKO611.1, AO may serve as an external stress which induced homologous recombination among three copies of Tn*A21*_partial and resulted in a large deletion (about 60 kbp) of a segment carrying ARGs. Homologous recombination between different copies of the same IS and transposons is frequently observed and may serve as an evolution mechanism (He et al., 2016).

Although pKO611.1 harbours *mcr-9.1*, which is typically found on HI2 plasmids (Li et al., 2020), *K. oxytoca* 611 and the *E. coli* transconjugant were not resistant to colistin. These results support studies which have shown that MCR 9.1 alone is not sufficient to confer colistin resistance (Carroll et al., 2019; Macesic et al., 2021). However, increased expression of MCR-9 was shown to contribute to colistin-resistance in *Enterobacter* strains, as removal of the *mcr-9* gene resulted in a 32-fold reduction in the MIC (Song et al., 2024). This suggest that *mcr-9* should still be closely monitored due to its high prevalence and circulation in healthcare settings (Song et al., 2024; Cui et al., 2024).

In addition to harbouring known ARGs, pKO611.1 carries two previously uncharacterized genes, *bla*_AKO-1_ and *tet*(E)*,* in the same resistance region. This resistance region was also found in multiple other plasmids in species such as *Aeromonas* spp., *K. pneumoniae*, and *R. ornithinolytica.* Furthermore, the resistance regions sequenced from pKO611.1, *K. pneumoniae* pNDM-KN, and *R. ornithinolytica* pWLK-NDM also carry a virulence factor, *mcpQ,* which encodes an MCP class of protein. MCPs play important roles in cell motility and adhesion, thereby contributing to bacterial survival and host colonization (Salah Ud-Din and Roujeinikova, 2017; Huang et al., 2017; Raghunandanan et al., 2025). The plasmid-encoded MCP identified in the opportunistic pathogen *Cronobacter sakazakii* (formerly *Enterobacter sakazakii*) has also been shown to contribute to biofilm formation, in addition to its role in host invasion (Choi et al., 2015). Although *K. oxytoca* 611 carries multiple other virulence genes on its chromosome, *mcpQ* has a high potential to be horizontally transferred to other bacteria.

The *bla*_AKO-1_ gene, encoding a class C β-lactamase, is postulated to have originally mobilised from *A. veroni,* a species ubiquitously found in aquatic environments and an emerging human opportunistic pathogen (Piotrowska and Popowska, 2014). *Aeromonas* spp. have previously been identified as the source of many ARGs including *mcr-3* and other β-lactamases (FOX and MOX) (McDonald et al., 2024; Ebmeyer et al., 2019a). Plasmid-encoded AmpC β-lactamases are frequently involved in β-lactam resistance in clinical strains, with the majority originating from chromosomes of *Enterobacter, Citrobacter* and *Aeromonas* spp (Ebmeyer et al., 2019b). Cloning and antibiotic susceptibility assays revealed that AKO-1 was able to confer resistance to all β-lactams tested including penicillins, all generations of cephalosporins and the monobactam, aztreonam, and β-lactams inhibitors including clavulanic acid and tazobactam in *E. coli* BL21(DE3) cells.

The *tet*(E) gene in pKO611.1 encodes a variant of the Tet(E) efflux pump, as determined by a comparison to the first class E Tet(A) identified from *E. coli* (Allard and Bertrand, 1993). Considerable sequence variation was observed in the C-terminal halves of these two proteins. Since the C-terminal region is involved more in substrate specificity (Paulsen et al., 1996) this variation could be indicative of an expanded substrate profile for Tet(E)-KO, which is able to confer tigecycline resistance. Tigecycline was developed to circumvent Tet efflux pump mediated resistance, but Tet efflux pumps have the potential to evolve and also confer tigecycline resistance (Linkevicius et al., 2016). Recently, new tetracycline efflux pump variants such as Tet(X3), Tet(Y) and Tet(L) were identified as contributing to tigecycline resistance (Wang et al., 2021a,b; Cheng et al., 2022), demonstrating the rapid evolution of these efflux pumps.

In addition to multiple ARGs found on resistance regions carried by pKO611.1, three prophage regions were also identified. One of these also carried ARGs, including *dfrA19, strA, strB*, and *mcr-9.1*. Interestingly, this prophage region was also identified in *Enterobacter adelaidei,* a new species recently isolated from wastewater sampled from a different hospital in Adelaide than the one from which *K. oxytoca* 611 was isolated (Siderius et al., 2024). This indicates that this prophage has circulated in the healthcare environment and may horizontally transfer ARGs through transduction. According to the NCBI database, this prophage region has been identified globally in other bacterial species including *Salmonella* spp. and *Citrobacter* spp. A recent study of prophages which assessed 38,605 bacterial genomes revealed that prophage-encoded ARGs found in human-impacted habitats were more abundant, diverse, and active (Liao et al., 2024). Additionally, antibiotic exposure facilitated the movement of ARGs between bacterial taxa and across habitats (Liao et al., 2024).

In this study, we isolated a novel hybrid self-transmissible mega plasmid, pKO611.1, that harbours an unprecedented 28 ARGs, from an XDR *K. oxytoca* isolated from hospital wastewater. We also identified and characterized two novel ARGs located on a resistance region within this novel plasmid. Both the resistance region and an associated prophage region carrying ARGs are globally distributed. Upon conjugation, pKO611.1 was able to confer high-level resistance to *E. coli*. The ease with which this plasmid transferred to *E. coli* where it conferred resistance to multiple antibiotics suggests that pKO611.1 may serve as a reservoir of ARGs for other pathogens. The identification of an XDR *K. oxytoca* strain harbouring a conjugative MDR plasmid in hospital wastewater is very concerning. This *K. oxytoca* could serve as a vessel for the transmission of MDR where environmental stressors, such as the high antibiotic content of hospital wastewater, could accelerate gene mobilisation and increase the pace of AMR development.

## Data statement

The Whole Genome Shotgun project of *K. oxytoca* 611 has been deposited at DDBJ/ENA/GenBank under the accession JAVRYI000000000. The version described in this paper is version JAVRYI020000000.

## CRediT authorship contribution statement

**Yu Wang:** Conceptualization, Validation, Formal analysis, Investigation, Data curation, Visualization, Writing – original draft, Writing – review & editing. **Sylvia A. Sapula:** Conceptualization, Validation, Formal analysis, Investigation, Data curation, Visualization, Supervision, Writing – review & editing. **Bradley J. Hart:** Methodology, Software, Formal analysis, Writing – review & editing. **Henrietta Venter:** Conceptualization, Resources, Data curation, Supervision, Project administration, Funding acquisition, Writing – review & editing.

## Declaration of competing interest

The authors declare that they have no known competing financial interests or personal relationships that could have appeared to influence the work reported in this paper.

## Appendix Supplementary materials

Image, application 1
