# Supplementary material for: A novel self-transmissible mega plasmid from extensively drug-resistant Klebsiella oxytoca carries multiple antimicrobial resistance genes and acts as a resistance reservoir
Source: Curr Res Microb Sci. 2026 Mar 24;10:100587. doi: 10.1016/j.crmicr.2026.100587 (PMC13085002; doi:10.1016/j.crmicr.2026.100587)
Supplement: Supplementary file 1 [file mmc1.docx]

# Extended data

**
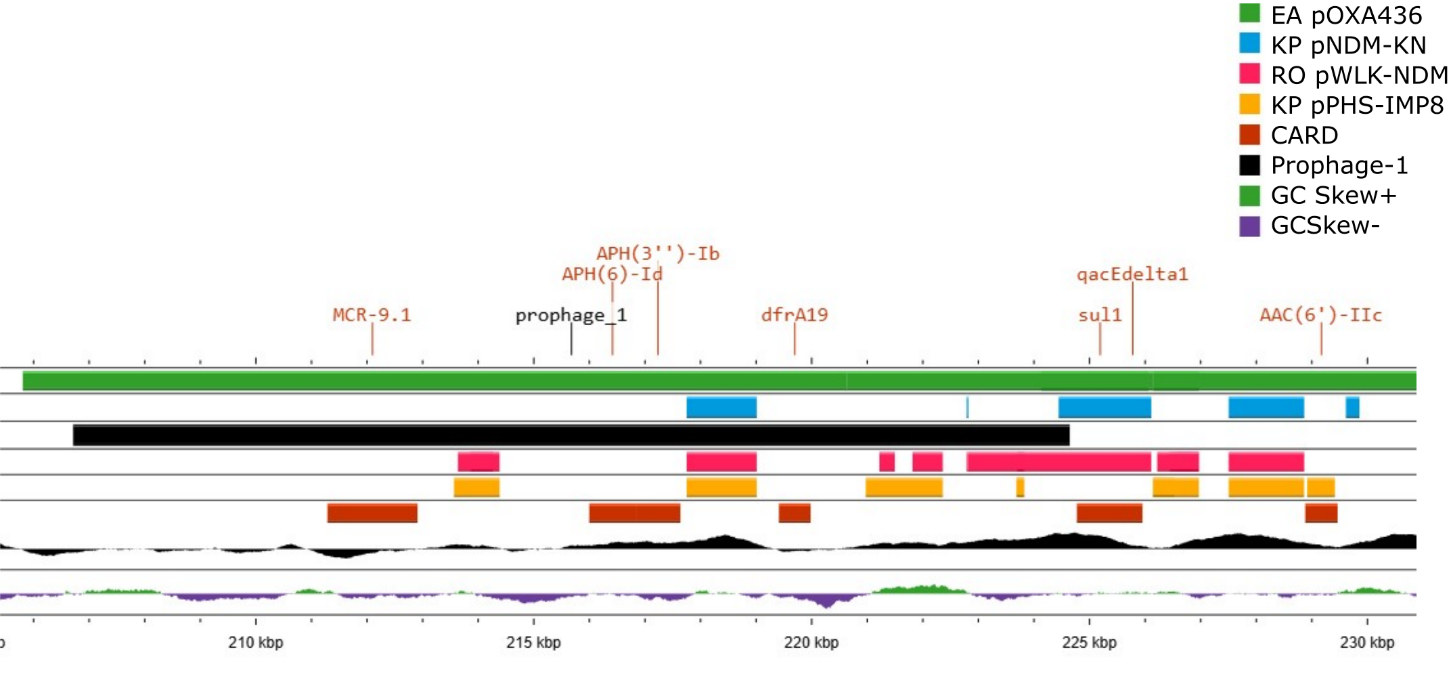
**

**Figure S1:** Prophage-1 region (black bar) carried by pKO611.1 with annotations.


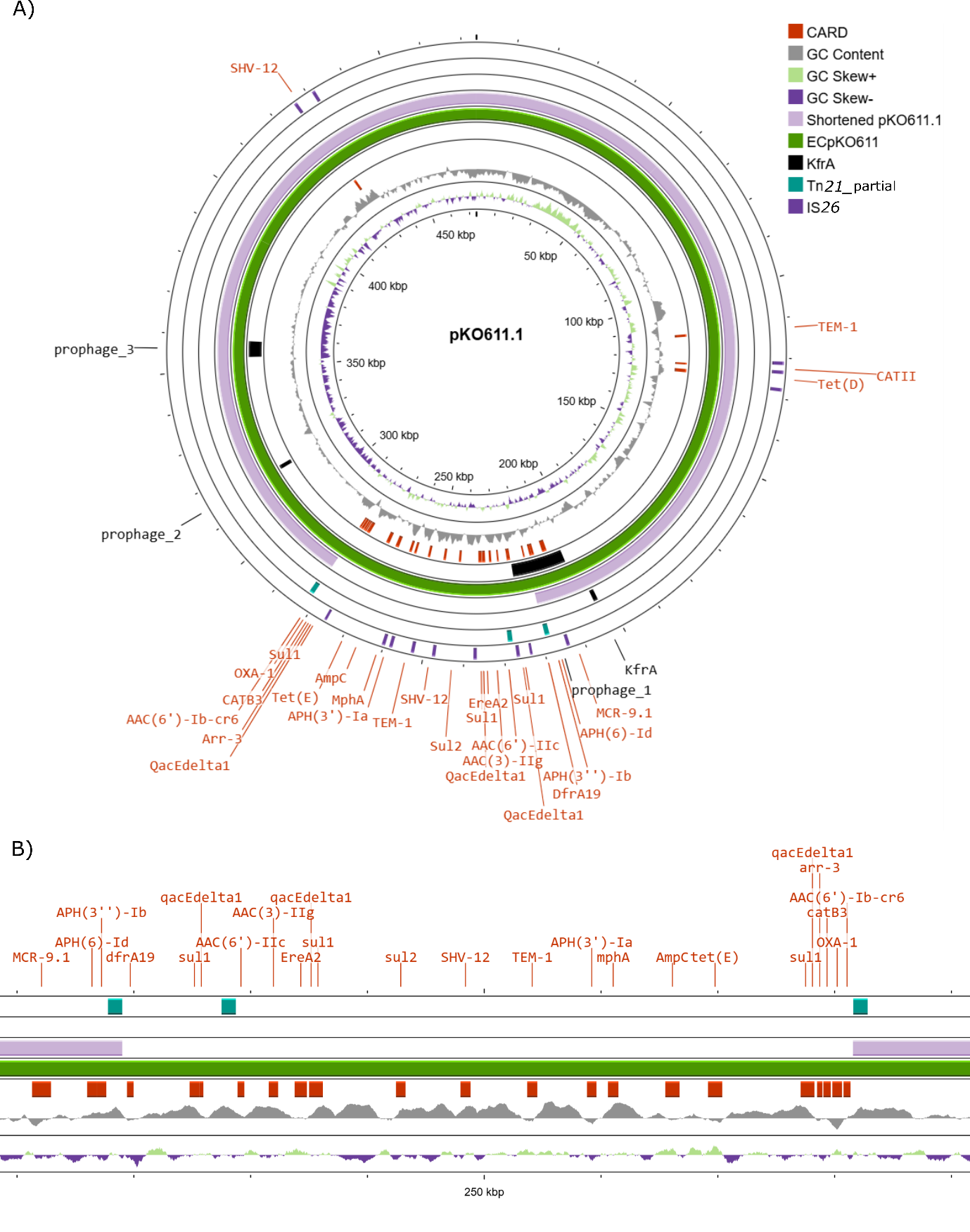


**Figure S2: Schematic representation of *E. coli* BW25113 pKO611.1 and *K. oxytoca* 611.1 (AO-treated). KfrA: DNA-binding protein. A)** Circular representation and **B)** liner representation of *E. coli* BW25113 transconjugant carrying pKO611.1, which is almost identical to *K. oxytoca* 611 (Identity: 99.99%). AO-treated *K. oxytoca* carried a truncated version of pKO611.1. Tn*21_partial* regions are also denoted.

**
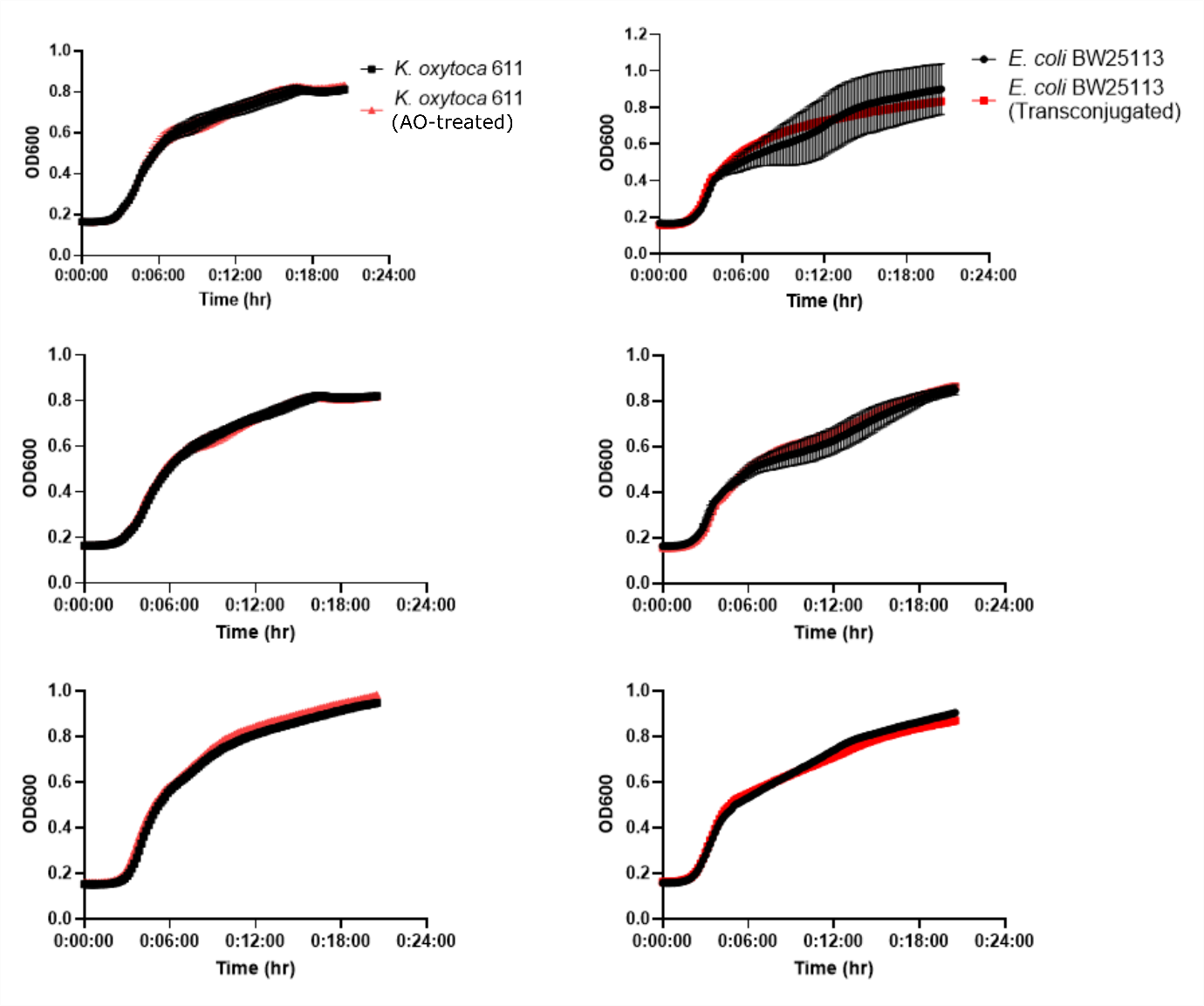
**

**Figure S3:** Growth profiles of bacteria in LB over 20 h. **Left:**  *K. oxytoca* 611 and *K. oxytoca* 611(AO-treated) **Right:** *E. coli* BW25113 and *E. coli* BW25113 (pKO611.1)


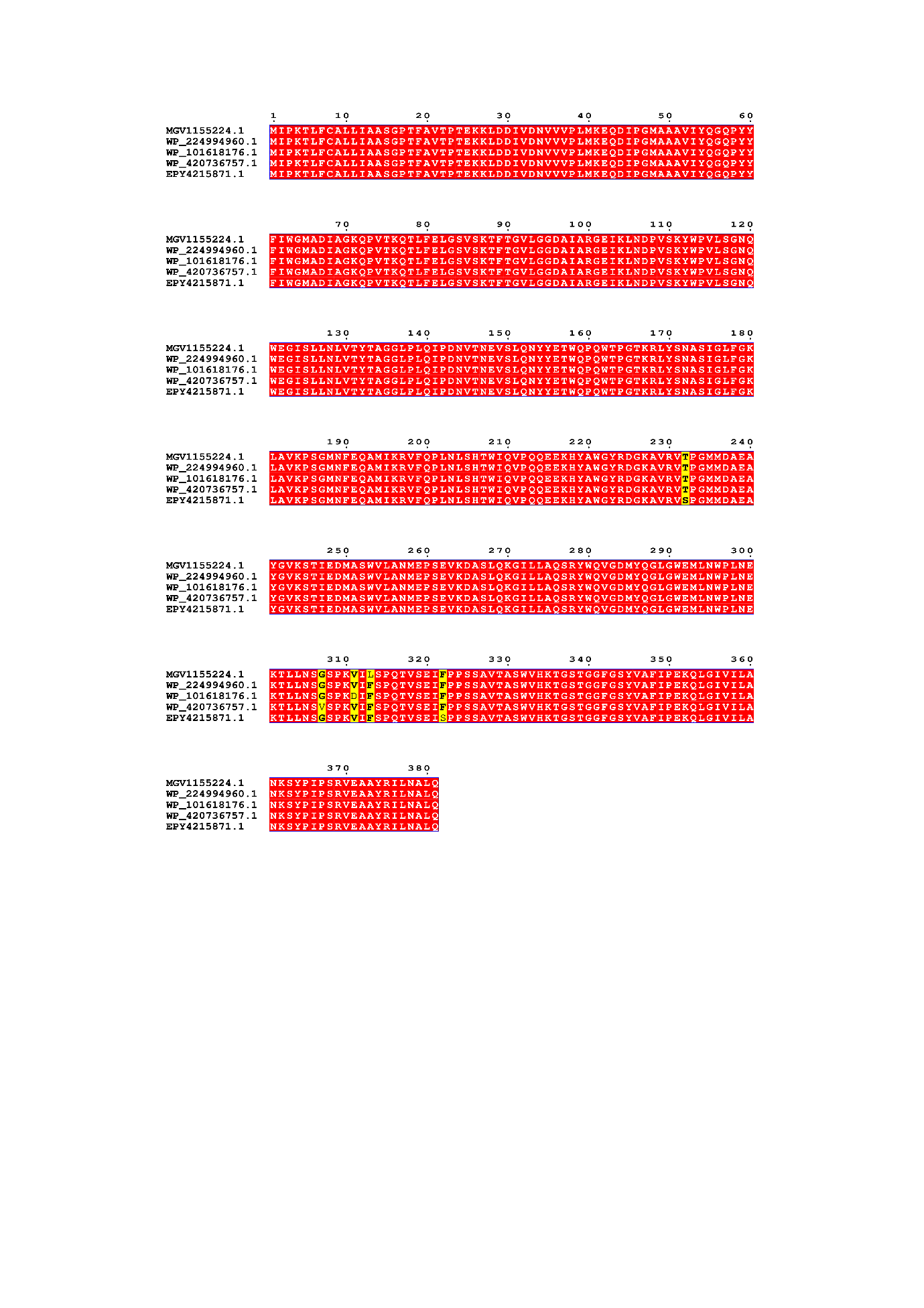


**Figure S4**: Protein alignment of five variants of the AKO family AmpC β-lactamases. Accession numbers are shown on the left. WP_224994960.1 is from *K. pneumoniae* (unclosed contig) and *Enterobacter kobei* (unclosed scaffold). WP_101618176.1 is from multiple species on plasmids as presented in **Figure 1**. It is also found on the chromosome of *Aeromonas veronii* (Genome assembly ASM4889804v1). WP_420736757.1 is from *Citrobacter freundii* on unclosed contig. EPY4215871.1 was from *Klebsiella quasipneumoniae.*


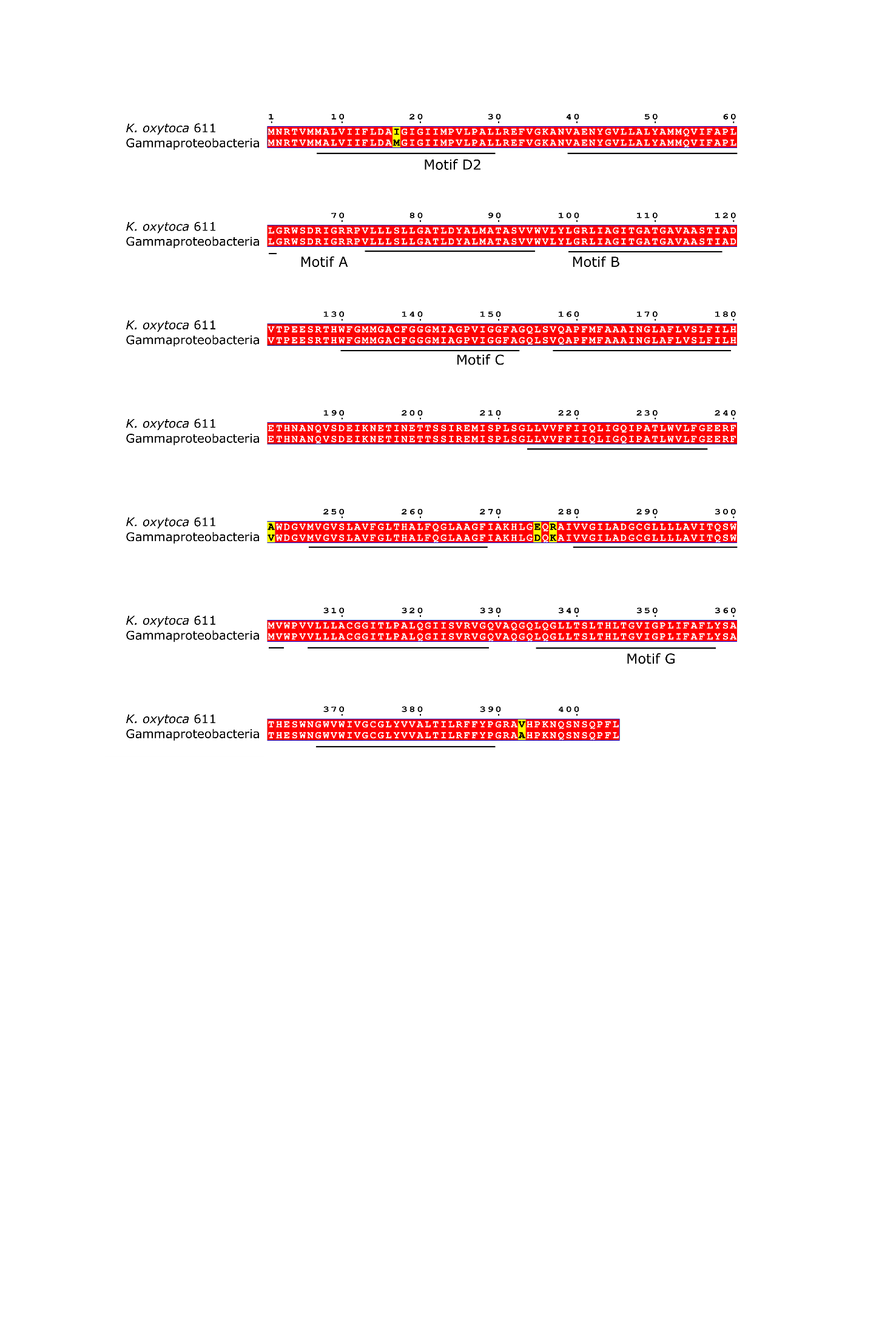


**Figure S5:** Protein alignment of pKO611.1 Tet(E) (Accession number: WP_017411290.1) and a Gammaproteobacteria Tet(E) (Accession number: WP_063856076.1). Transmembrane helices are underlined. Motif D2: lgxxxxxPvxP, Motif A: GxLaDrxGrkxxl, Motif B: lxxxRxxqGxgaa, Motif C: gxxxGPxxGGxl, Motif G: GxxxGPL.

**Table S1: Antibiotic resistance proteins encoded on pKO611.1.**

|  | **Start** | **Stop** | **Strand** | **Proteins** | **Classification** | **Substrate** |
| --- | --- | --- | --- | --- | --- | --- |
| 1 | 112091 | 112951 | -1 | TEM-1 | narrow-spectrum β-lactamases | β-lactams |
| 2 | 122308 | 122949 | 1 | CATII | Chloramphenicol resistance protein | Chloramphenicol |
| 3 | 124561 | 125745 | 1 | Tet(D) | Tetracycline efflux pump | Tetracyclines |
| 4 | 211289 | 212908 | 1 | MCR9.1 | mobile colistin resistance | colistin |
| 5 | 215999 | 216835 | -1 | APH(6)-Id | aminoglycoside phosphotransferase | aminoglycoside |
| 6 | 216835 | 217638 | -1 | APH(3′′)-Ib | aminoglycoside phosphotransferase | aminoglycoside |
| 7 | 219412 | 219981 | -1 | DfrA19 | trimethoprim resistant dihydrofolate reductase dfr | trimethoprim |
| 8 | 224772 | 225611 | -1 | Sul1 | sulfonamide resistant dihydropteroate synthase | sulfonamides |
| 9 | 225605 | 225952 | -1 | QacEΔ1 | Antiseptic-resistance protein | quaternary ammonium compounds |
| 10 | 228882 | 229463 | 1 | AAC(6')-IIc | aminoglycoside phosphotransferase | aminoglycoside |
| 11 | 231548 | 232357 | 1 | AAC(3)-IIg | aminoglycoside phosphotransferase | aminoglycoside |
| 12 | 233773 | 234819 | 1 | EreA2 | erythromycin esterase | erythromycin |
| 13 | 235002 | 235349 | 1 | QacEΔ1 | Antiseptic-resistance protein | quaternary ammonium compounds |
| 14 | 235343 | 236182 | 1 | Sul1 | sulfonamide resistant dihydropteroate synthase | sulfonamides |
| 15 | 242448 | 243263 | 1 | Sul2 | sulfonamide resistant dihydropteroate synthase | sulfonamides |
| 16 | 247963 | 248823 | 1 | SHV-12 | Extended-spectrum β-lactamase | β-lactams |
| 17 | 253677 | 253677 | 1 | TEM-1 | narrow-spectrum β-lactamases | β-lactams |
| 18 | 258796 | 259611 | -1 | APH(3')-Ia | aminoglycoside phosphotransferase | aminoglycoside |
| 19 | 260588 | 261493 | 1 | MPH(2')-I | macrolide 2'-phosphotransferase I | macrolides |
| 20 | 265497 | 266729 | 1 | AmpC | AmpC β-lactamases | β-lactams |
| 21 | 269155 | 270372 | 1 | Tet(E) | Tetracycline efflux pump | Tetracyclines |
| 22 | 277094 | 277933 | -1 | Sul1 | sulfonamide resistant dihydropteroate synthase | sulfonamides |
| 23 | 277927 | 278274 | -1 | QacEΔ1 | Antiseptic-resistance protein | antiseptics |
| 24 | 278497 | 278949 | -1 | Arr-3 | ribosyltransferase | Rifamycin |
| 25 | 279034 | 279666 | -1 | CATB3 | Chloramphenicol resistance protein | Chloramphenicol |
| 26 | 279804 | 280634 | -1 | OXA-1 | OXA-type β-lactamase | β-lactams |
| 27 | 280764 | 281364 | -1 | AAC(6')-Ib-cr6 | fluoroquinolone-acetylating aminoglycoside acetyltransferase | fluoroquinolone and aminoglycoside |
| 28 | 426750 | 427610 | 1 | SHV-12 | Extended-spectrum β-lactamase | β-lactams |

**Table S2: Antibiotic resistance proteins encoded on chromosome of *K. oxytoca* 611.**

|  | **Start** | **Stop** | **Strand** | **Proteins** | **Classification** | **Substrate** |
| --- | --- | --- | --- | --- | --- | --- |
| 1 | 212540 | 214228 | 1 | EptB | pmr phosphoethanolamine transferase | peptide antibiotics |
| 2 | 318476 | 319459 | 1 | PmrF | pmr phosphoethanolamine transferase | peptide antibiotic |
| 3 | 322340 | 323995 | 1 | ArnT | pmr phosphoethanolamine transferase | pmr phosphoethanolamine transferase |
| 4 | 430613 | 431245 | -1 | CRP | β-lactamase | β-lactams |
| 5 | 1247184 | 1247369 | 1 | RsmA | resistance-nodulation-cell division (RND) antibiotic efflux pump | phenicol antibiotic, diaminopyrimidine antibiotic, fluoroquinolone antibiotic |
| 6 | 1255369 | 1256907 | -1 | KpnH | major facilitator superfamily (MFS) antibiotic efflux pump | peptide antibiotic, penicillin β-lactam, cephalosporin, aminoglycoside antibiotic, carbapenem, fluoroquinolone antibiotic, macrolide antibiotic |
| 7 | 1256923 | 1258095 | -1 | KpnG | major facilitator superfamily (MFS) antibiotic efflux pump | peptide antibiotic, penicillin β-lactam, cephalosporin, aminoglycoside antibiotic, carbapenem, fluoroquinolone antibiotic, macrolide antibiotic |
| 8 | 1258229 | 1258759 | -1 | EmrR | major facilitator superfamily (MFS) antibiotic efflux pump | fluoroquinolone antibiotic |
| 9 | 1505204 | 1508317 | -1 | AcrD | resistance-nodulation-cell division (RND) antibiotic efflux pump | aminoglycoside antibiotic |
| 10 | 1878283 | 1879005 | -1 | BaeR | resistance-nodulation-cell division (RND) antibiotic efflux pump | aminocoumarin antibiotic, aminoglycoside antibiotic |
| 11 | 1881945 | 1885022 | -1 | MdtC | resistance-nodulation-cell division (RND) antibiotic efflux pump | aminocoumarin antibiotic |
| 12 | 1885023 | 1888145 | -1 | MdtB | resistance-nodulation-cell division (RND) antibiotic efflux pump | aminocoumarin antibiotic |
| 13 | 2593802 | 2594164 | 1 | KpnE | small multidrug resistance (SMR) antibiotic efflux pump | peptide antibiotic, cephalosporin, disinfecting agents and antiseptics, rifamycin antibiotic, tetracycline antibiotic, aminoglycoside antibiotic, macrolide antibiotic |
| 14 | 2594151 | 2594480 | 1 | KpnF | small multidrug resistance (SMR) antibiotic efflux pump | peptide antibiotic, cephalosporin, disinfecting agents and antiseptics, rifamycin antibiotic, tetracycline antibiotic, aminoglycoside antibiotic, macrolide antibiotic |
| 15 | 2665814 | 2666194 | -1 | MarA | AcrAB regulator (activator) | tetracycline antibiotic, penicillin β-lactam, cephalosporin, disinfecting agents and antiseptics, phenicol antibiotic, rifamycin antibiotic, glycylcycline, carbapenem, monobactam, fluoroquinolone antibiotic |
| 16 | 3139938 | 3143090 | -1 | OqxB | resistance-nodulation-cell division (RND) antibiotic efflux pump | tetracycline antibiotic, nitrofuran antibiotic, diaminopyrimidine antibiotic, glycylcycline, fluoroquinolone antibiotic |
| 17 | 3143114 | 3144289 | -1 | OqxA | resistance-nodulation-cell division (RND) antibiotic efflux pump | tetracycline antibiotic, nitrofuran antibiotic, diaminopyrimidine antibiotic, glycylcycline, fluoroquinolone antibiotic |
| 18 | 3643179 | 3644249 | 1 | OmpA | porin | peptide antibiotic |
| 19 | 3685608 | 3687356 | -1 | MsbA | ATP-binding cassette (ABC) antibiotic efflux pump | nitroimidazole antibiotic |
| 20 | 4225977 | 4226849 | 1 | OXY-2-11 | β-lactamase | penicillin β-lactam, cephalosporin, monobactam |
| 21 | 4318731 | 4321877 | 1 | AcrB | resistance-nodulation-cell division (RND) antibiotic efflux pump | tetracycline antibiotic, penicillin β-lactam, cephalosporin, disinfecting agents and antiseptics, phenicol antibiotic, rifamycin antibiotic, glycylcycline, fluoroquinolone antibiotic |
| 22 | 4477317 | 4478414 | 1 | VanG | Van ligase, glycopeptide resistance gene cluster | glycopeptide antibiotic |
| 23 | 4797062 | 4798012 | -1 | LeuO | major facilitator superfamily (MFS) antibiotic efflux pump | nucleoside antibiotic, disinfecting agents and antiseptics |
| 24 | 5042218 | 5042661 | -1 | FosA5 | fosfomycin thiol transferase | phosphonic acid antibiotic, aminoglycoside antibiotic, fluoroquinolone antibiotic |
| 25 | 3757 | 6171 | 1 | GyrB | DNA gyrase mutation | Fluoroquinolones |
| 26 | 42194 | 43585 | 1 | UhpT | antibiotic-resistant UhpT | Fosfomycin |
| 27 | 446244 | 447428 | 1 | EF-Tu | mutation of elongation factor | Pulvomycin |
| 28 | 1701949 | 1704582 | 1 | GyrA | DNA gyrase mutation | Fluoroquinolones |
| 29 | 4789095 | 4790861 | -1 | PBP3 | Penicillin-binding protein mutations | β-lactams (cephalosporin and penicillin) |
| 30 | 5497526 | 5498710 | -1 | EF-Tu | mutation of elongation factor | Pulvomycin |
| 31 | 2666212 | 2666646 | -1 | MarR | AcrAB regulator (repressor) | ciprofloxacin and tetracycline |

**Table S3 Virulence proteins encoded on chromosome of *K. oxytoca* 611 from the virulence factors database BLAST search (E value < 2e-05)**

| VFDB accession number | Name of the virulence factor | Length | Position of the blast result with highest identities/length | Identities | E value |
| --- | --- | --- | --- | --- | --- |
| VFG049144 | acriflavine resistance protein B | 3147 | 4318731-4321877 | 2737/3147 (86%) | 0 |
| VFG000363 | yersiniabactin biosynthetic protein Irp2 | 6108 | 2824678- 2827686 | 2612/3009 (86%) | 0 |
| VFG044328 | yersiniabactin non-ribosomal peptide synthetase HMWP2 | 6108 | 2824678- 2827686 | 2609/3009 (86%) | 0 |
| VFG000358 | pesticin/yersiniabactin receptor protein | 2022 | 2806702-  2808723 | 1809/2022 (89%) | 0 |
| VFG044333 | yersiniabactin receptor FyuA | 2022 | 2806702- 2808723 | 1808/2022 (89%) | 0 |
| VFG000362 | yersiniabactin biosynthetic protein Irp1 | 9492 | 2817992- 2820404 | 2094/2414 (86%) | 0 |
| VFG044329 | (irp1) yersiniabactin polyketide synthase HMWP1 | 9492 | 2817992-  2820404 | 2093/2414 (86%) | 0 |
| VFG049120 | (allB) allantoinase | 1362 | 4269994-  4271355 | 1226/1362 (90%) | 0 |
| VFG043625 | (mrkC) fimbrial biogenesis outer membrane usher protein mrkC precursor | 2487 | 966360-  968385 | 1705/2026 (84%) | 0 |
| VFG049122 | (allC) allantoate amidohydrolase | 1236 | 4265117-  4266348 | 1104/1232 (89%) | 0 |

**Table S4. Virulence factors encoded on pKO611.1 from the virulence factors database BLAST search (E value < 2e-05)**

| VFDB accession number | Name of the virulence factor | Length | Position of the blast result with highest identities/length | Identities | E value |
| --- | --- | --- | --- | --- | --- |
| VFG043209 | methyl-accepting chemotaxis protein CheD | 1674 | 263738-263777 | 38/40 (95%) | 2e-07 |
